# Ethyl 3-(3-eth­oxy-2-hydroxy­benzyl­idene)carbazate

**DOI:** 10.1107/S1600536809044626

**Published:** 2009-10-31

**Authors:** Yu-Feng Li, Hai-Xing Liu, Fang-Fang Jian

**Affiliations:** a Microscale Science Institute, Department of Chemistry and Chemical Engineering, Weifang University, Weifang 261061, People’s Republic of China; b Microscale Science Institute, Weifang University, Weifang 261061, People’s Republic of China

## Abstract

In the title compound, C_12_H_16_N_2_O_4_, an intra­molecular O—H⋯O hydrogen bond occurs. In the crystal, mol­ecules are linked by N—H⋯O hydrogen bonds, forming chains propagating in the [010] direction.

## Related literature

For background to Schiff bases, see: Cimerman *et al.* (1997 ▶).
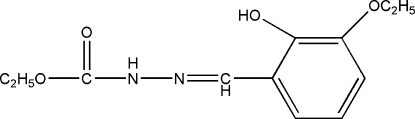


## Experimental

### 

#### Crystal data

C_12_H_16_N_2_O_4_
*M* *_r_* = 252.27
Orthorhombic, 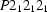

*a* = 7.1140 (14) Å
*b* = 9.6010 (19) Å
*c* = 18.570 (4) Å
*V* = 1268.4 (4) Å^3^
*Z* = 4
Mo *K*α radiation
μ = 0.10 mm^−1^
*T* = 293 K
0.22 × 0.20 × 0.19 mm

#### Data collection

Bruker SMART CCD diffractometer
Absorption correction: none
11976 measured reflections
2917 independent reflections
2680 reflections with *I* > 2σ(*I*)
*R*_int_ = 0.043

#### Refinement

*R*[*F* ^2^ > 2σ(*F* ^2^)] = 0.035
*wR*(*F* ^2^) = 0.090
*S* = 1.07
2917 reflections
163 parameters
H-atom parameters constrained
Δρ_max_ = 0.14 e Å^−3^
Δρ_min_ = −0.18 e Å^−3^

### 

Data collection: *SMART* (Bruker, 1997 ▶); cell refinement: *SAINT* (Bruker, 1997 ▶); data reduction: *SAINT*; program(s) used to solve structure: *SHELXS97* (Sheldrick, 2008 ▶); program(s) used to refine structure: *SHELXL97* (Sheldrick, 2008 ▶); molecular graphics: *SHELXTL* (Sheldrick, 2008 ▶); software used to prepare material for publication: *SHELXTL*.

## Supplementary Material

Crystal structure: contains datablocks global, I. DOI: 10.1107/S1600536809044626/hb5155sup1.cif

Structure factors: contains datablocks I. DOI: 10.1107/S1600536809044626/hb5155Isup2.hkl

Additional supplementary materials:  crystallographic information; 3D view; checkCIF report

## Figures and Tables

**Table 1:** Hydrogen-bond geometry (Å, °)

*D*—H⋯*A*	*D*—H	H⋯*A*	*D*⋯*A*	*D*—H⋯*A*
O4—H4*A*⋯N2	0.82	1.91	2.6290 (15)	145
N1—H1*A*⋯O2^i^	0.86	2.31	2.9633 (15)	132

Symmetry code: (i) 
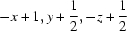
.

**Fig. 1.**
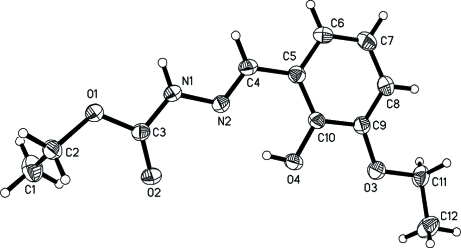
The molecular structure of (I) showing 30% probability displacement ellipsoids.

## supplementary crystallographic information

### Experimental

A mixture of 3-ethoxy-2-hydroxybenzaldehyde (0.1 mol), and ethyl carbazate (0.1 mol) was stirred in refluxing ethanol (20 ml) for 4 h to afford the title compound (0.082 mol, yield 82%).  Colourless blocks of (I) were obtained by recrystallization from ethanol at room temperature.

### Refinement

Refinement of the Flack absolute structure parameter was indeterminate.

H atoms were fixed geometrically (C—H = 0.93–0.97Å, O—H = 0.82Å, N—H = 0.86Å) and refined as riding with *U*_iso_(H) = 1.2–1.5*U*_eq_(carrier).

### Crystal data

**Table:**

C_12_H_16_N_2_O_4_	*F*(000) = 536
*M**_r_* = 252.27	*D*_x_ = 1.321 Mg m^−^^3^
Orthorhombic, *P*2_1_2_1_2_1_	Mo *K*α radiation, λ = 0.71073 Å
Hall symbol: P 2ac 2ab	Cell parameters from 1982 reflections
*a* = 7.1140 (14) Å	θ = 3.5–27.3°
*b* = 9.6010 (19) Å	µ = 0.10 mm^−^^1^
*c* = 18.570 (4) Å	*T* = 293 K
*V* = 1268.4 (4) Å^3^	Block, colourless
*Z* = 4	0.22 × 0.20 × 0.19 mm

### Data collection

**Table:**

Bruker SMART CCD diffractometer	2680 reflections with *I* > 2σ(*I*)
Radiation source: fine-focus sealed tube	*R*_int_ = 0.043
graphite	θ_max_ = 27.5°, θ_min_ = 3.1°
ω scans	*h* = −8→9
11976 measured reflections	*k* = −12→12
2917 independent reflections	*l* = −23→24

### Refinement

**Table:**

Refinement on *F*^2^	Primary atom site location: structure-invariant direct methods
Least-squares matrix: full	Secondary atom site location: difference Fourier map
*R*[*F*^2^ > 2σ(*F*^2^)] = 0.035	Hydrogen site location: inferred from neighbouring sites
*wR*(*F*^2^) = 0.090	H-atom parameters constrained
*S* = 1.07	*w* = 1/[σ^2^(*F*_o_^2^) + (0.0474*P*)^2^ + 0.0849*P*] where *P* = (*F*_o_^2^ + 2*F*_c_^2^)/3
2917 reflections	(Δ/σ)_max_ < 0.001
163 parameters	Δρ_max_ = 0.14 e Å^−^^3^
0 restraints	Δρ_min_ = −0.18 e Å^−^^3^

### Special details

**Table:**

Geometry. All e.s.d.'s (except the e.s.d. in the dihedral angle between two l.s. planes) are estimated using the full covariance matrix. The cell e.s.d.'s are taken into account individually in the estimation of e.s.d.'s in distances, angles and torsion angles; correlations between e.s.d.'s in cell parameters are only used when they are defined by crystal symmetry. An approximate (isotropic) treatment of cell e.s.d.'s is used for estimating e.s.d.'s involving l.s. planes.
Refinement. Refinement of *F*^2^ against ALL reflections. The weighted *R*-factor *wR* and goodness of fit *S* are based on *F*^2^, conventional *R*-factors *R* are based on *F*, with *F* set to zero for negative *F*^2^. The threshold expression of *F*^2^ > σ(*F*^2^) is used only for calculating *R*-factors(gt) *etc*. and is not relevant to the choice of reflections for refinement. *R*-factors based on *F*^2^ are statistically about twice as large as those based on *F*, and *R*- factors based on ALL data will be even larger.

### Fractional atomic coordinates and isotropic or equivalent isotropic displacement parameters (Å^2^)

**Table:**

	*x*	*y*	*z*	*U*_iso_*/*U*_eq_	
O1	0.32636 (13)	0.00340 (9)	0.17092 (5)	0.0507 (3)	
N2	0.73581 (14)	−0.03948 (10)	0.26990 (6)	0.0400 (2)	
O4	0.90478 (14)	−0.23476 (9)	0.34513 (6)	0.0514 (3)	
H4A	0.8193	−0.2008	0.3212	0.077*	
N1	0.59340 (15)	0.01343 (11)	0.22830 (6)	0.0447 (3)	
H1A	0.5964	0.0986	0.2140	0.054*	
O2	0.43343 (15)	−0.19175 (9)	0.22560 (6)	0.0530 (3)	
C3	0.44812 (18)	−0.07057 (13)	0.21020 (7)	0.0398 (3)	
O3	1.19430 (15)	−0.31435 (11)	0.41917 (6)	0.0563 (3)	
C10	1.04046 (17)	−0.13798 (13)	0.35560 (7)	0.0379 (3)	
C4	0.87138 (19)	0.04316 (13)	0.28510 (7)	0.0418 (3)	
H4B	0.8679	0.1344	0.2684	0.050*	
C5	1.03034 (18)	−0.00269 (13)	0.32791 (7)	0.0396 (3)	
C8	1.3410 (2)	−0.08581 (17)	0.40856 (8)	0.0522 (3)	
H8A	1.4449	−0.1125	0.4356	0.063*	
C9	1.1983 (2)	−0.17984 (14)	0.39607 (7)	0.0433 (3)	
C11	1.3491 (2)	−0.36144 (18)	0.46182 (8)	0.0569 (4)	
H11A	1.3584	−0.3070	0.5057	0.068*	
H11B	1.4657	−0.3519	0.4352	0.068*	
C6	1.1784 (2)	0.08977 (15)	0.34131 (8)	0.0500 (3)	
H6A	1.1732	0.1799	0.3231	0.060*	
C2	0.1594 (2)	−0.06859 (15)	0.14570 (9)	0.0503 (3)	
H2B	0.0623	−0.0007	0.1351	0.060*	
H2C	0.1131	−0.1291	0.1836	0.060*	
C7	1.3308 (2)	0.04877 (18)	0.38097 (9)	0.0583 (4)	
H7A	1.4282	0.1112	0.3895	0.070*	
C12	1.3135 (3)	−0.51120 (19)	0.47940 (11)	0.0702 (5)	
H12A	1.4159	−0.5468	0.5076	0.105*	
H12B	1.3032	−0.5638	0.4356	0.105*	
H12C	1.1987	−0.5191	0.5063	0.105*	
C1	0.1972 (3)	−0.1532 (2)	0.08008 (10)	0.0710 (5)	
H1B	0.0839	−0.1994	0.0654	0.106*	
H1C	0.2922	−0.2212	0.0905	0.106*	
H1D	0.2399	−0.0933	0.0420	0.106*	

### Atomic displacement parameters (Å^2^)

**Table:**

	*U*^11^	*U*^22^	*U*^33^	*U*^12^	*U*^13^	*U*^23^
O1	0.0523 (5)	0.0346 (5)	0.0654 (6)	0.0008 (4)	−0.0193 (5)	0.0033 (4)
N2	0.0422 (5)	0.0344 (5)	0.0433 (5)	0.0040 (4)	−0.0032 (4)	0.0007 (4)
O4	0.0488 (5)	0.0345 (5)	0.0708 (6)	−0.0054 (4)	−0.0190 (5)	0.0043 (4)
N1	0.0486 (6)	0.0311 (5)	0.0545 (6)	0.0019 (4)	−0.0116 (5)	0.0051 (4)
O2	0.0618 (6)	0.0288 (4)	0.0683 (6)	0.0011 (4)	−0.0139 (5)	0.0029 (4)
C3	0.0454 (6)	0.0310 (5)	0.0429 (6)	0.0050 (5)	−0.0035 (6)	−0.0037 (5)
O3	0.0554 (6)	0.0443 (5)	0.0693 (6)	0.0021 (4)	−0.0214 (5)	0.0068 (4)
C10	0.0393 (6)	0.0355 (6)	0.0387 (6)	−0.0011 (5)	−0.0014 (5)	−0.0041 (5)
C4	0.0484 (6)	0.0325 (6)	0.0445 (6)	0.0012 (5)	0.0015 (6)	0.0015 (5)
C5	0.0413 (6)	0.0398 (6)	0.0376 (6)	−0.0017 (5)	0.0014 (5)	−0.0025 (5)
C8	0.0446 (7)	0.0583 (9)	0.0535 (8)	−0.0041 (6)	−0.0122 (7)	−0.0008 (6)
C9	0.0457 (7)	0.0416 (7)	0.0426 (6)	0.0011 (5)	−0.0049 (6)	−0.0016 (5)
C11	0.0536 (8)	0.0620 (9)	0.0550 (8)	0.0109 (7)	−0.0132 (7)	0.0057 (7)
C6	0.0533 (8)	0.0414 (7)	0.0554 (8)	−0.0104 (6)	−0.0007 (7)	0.0036 (6)
C2	0.0455 (7)	0.0405 (7)	0.0647 (8)	0.0012 (6)	−0.0117 (7)	−0.0035 (6)
C7	0.0506 (8)	0.0592 (9)	0.0650 (9)	−0.0189 (7)	−0.0082 (7)	−0.0003 (7)
C12	0.0665 (10)	0.0650 (10)	0.0791 (11)	0.0153 (9)	−0.0034 (9)	0.0201 (9)
C1	0.0691 (10)	0.0819 (12)	0.0618 (9)	0.0041 (10)	−0.0130 (9)	−0.0153 (9)

### Geometric parameters (Å, °)

**Table:**

O1—C3	1.3367 (15)	C8—C7	1.392 (2)
O1—C2	1.4516 (17)	C8—H8A	0.9300
N2—C4	1.2803 (17)	C11—C12	1.496 (3)
N2—N1	1.3715 (14)	C11—H11A	0.9700
O4—C10	1.3539 (15)	C11—H11B	0.9700
O4—H4A	0.8200	C6—C7	1.368 (2)
N1—C3	1.3533 (16)	C6—H6A	0.9300
N1—H1A	0.8600	C2—C1	1.489 (2)
O2—C3	1.2026 (15)	C2—H2B	0.9700
O3—C9	1.3611 (17)	C2—H2C	0.9700
O3—C11	1.4299 (17)	C7—H7A	0.9300
C10—C5	1.3988 (18)	C12—H12A	0.9600
C10—C9	1.4095 (18)	C12—H12B	0.9600
C4—C5	1.4508 (18)	C12—H12C	0.9600
C4—H4B	0.9300	C1—H1B	0.9600
C5—C6	1.3998 (18)	C1—H1C	0.9600
C8—C9	1.3783 (19)	C1—H1D	0.9600
			
C3—O1—C2	116.95 (10)	O3—C11—H11B	110.3
C4—N2—N1	116.81 (10)	C12—C11—H11B	110.3
C10—O4—H4A	109.5	H11A—C11—H11B	108.5
C3—N1—N2	118.90 (10)	C7—C6—C5	120.62 (13)
C3—N1—H1A	120.5	C7—C6—H6A	119.7
N2—N1—H1A	120.5	C5—C6—H6A	119.7
O2—C3—O1	125.97 (12)	O1—C2—C1	112.09 (13)
O2—C3—N1	125.72 (12)	O1—C2—H2B	109.2
O1—C3—N1	108.29 (10)	C1—C2—H2B	109.2
C9—O3—C11	117.30 (12)	O1—C2—H2C	109.2
O4—C10—C5	123.22 (11)	C1—C2—H2C	109.2
O4—C10—C9	116.66 (11)	H2B—C2—H2C	107.9
C5—C10—C9	120.12 (11)	C6—C7—C8	120.42 (13)
N2—C4—C5	121.33 (11)	C6—C7—H7A	119.8
N2—C4—H4B	119.3	C8—C7—H7A	119.8
C5—C4—H4B	119.3	C11—C12—H12A	109.5
C10—C5—C6	119.00 (12)	C11—C12—H12B	109.5
C10—C5—C4	121.56 (11)	H12A—C12—H12B	109.5
C6—C5—C4	119.44 (12)	C11—C12—H12C	109.5
C9—C8—C7	120.52 (13)	H12A—C12—H12C	109.5
C9—C8—H8A	119.7	H12B—C12—H12C	109.5
C7—C8—H8A	119.7	C2—C1—H1B	109.5
O3—C9—C8	125.71 (12)	C2—C1—H1C	109.5
O3—C9—C10	114.97 (12)	H1B—C1—H1C	109.5
C8—C9—C10	119.32 (12)	C2—C1—H1D	109.5
O3—C11—C12	107.13 (14)	H1B—C1—H1D	109.5
O3—C11—H11A	110.3	H1C—C1—H1D	109.5
C12—C11—H11A	110.3		

### Hydrogen-bond geometry (Å, °)

**Table:**

*D*—H···*A*	*D*—H	H···*A*	*D*···*A*	*D*—H···*A*
O4—H4A···N2	0.82	1.91	2.6290 (15)	145
N1—H1A···O2^i^	0.86	2.31	2.9633 (15)	132

Symmetry codes: (i) −*x*+1, *y*+1/2, −*z*+1/2.
